# Lead detoxification of edible fungi *Auricularia auricula* and *Pleurotus ostreatus*: the purification of the chelation substances and their effects on rats

**DOI:** 10.3389/fnut.2023.1162110

**Published:** 2023-04-20

**Authors:** Weiwei Zhang, Xiaojie Zheng, Xiangdong Chen, Xuezhen Jiang, Hexiang Wang, Guoqing Zhang

**Affiliations:** ^1^Institute of Medicinal Plant Development, Chinese Academy of Medical Sciences & Peking Union Medical College, Beijing, China; ^2^State Key Laboratory for Agrobiotechnology, College of Biological Sciences, China Agricultural University, Beijing, China; ^3^College of Plant Science and Technology, Beijing Key Laboratory for Agricultural Application and New Technique, Beijing University of Agriculture, Beijing, China

**Keywords:** polysaccharide-peptide, lead clearance, chelation, antidotes, edible fungi

## Abstract

Lead is a global pollutant that causes widespread concern. When a lead enters the body, it is distributed throughout the body and accumulates in the brain, bone, and soft tissues such as the kidney, liver, and spleen. Chelators used for lead poisoning therapy all have side effects to some extent and other drawbacks including high cost. Exploration and utilization of natural antidotes become necessary. To date, few substances originating from edible fungi that are capable of adsorbing lead have been reported. In this study, we found that two commonly eaten mushrooms *Auricularia auricula* and *Pleurotus ostreatus* exhibited lead adsorption capacity. *A. auricula* active substance (AAAS) and *P. ostreatus* active substance (POAS) were purified by hot-water extraction, ethanol precipitation from its fruiting bodies followed by ion exchange chromatography, ultrafiltration, and gel filtration chromatography, respectively. AAAS was 3.6 kDa, while POAS was 4.9 kDa. They were both constituted of polysaccharides and peptides. The peptide sequences obtained by liquid chromatography combined with tandem mass spectrometry (LC-MS/MS) proved that they were rich in amino acids with side chain groups such as hydroxyl, carboxyl, carbonyl, sulfhydryl, and amidogen. Two rat models were established, but only a chronic lead-induced poisoning model was employed to determine the detoxification of AAAS/POAS and their fruiting body powder. For rats receiving continuous lead treatment, either AAAS or POAS could reduce the lead levels in the blood. They also promoted the elimination of the burden of lead in the spleen and kidney. The fruiting bodies were also proved to have lead detoxification effects. This is the first study to identify new functions of *A. auricula* and *P. ostreatus* in reducing lead toxicity and to provide dietary strategies for the treatment of lead toxicity.

## Introduction

Lead, a well-known toxic heavy metal, is widely distributed in soil, water, and air. Various industrial activities contain lead or lead-based components, such as lead acid battery manufacturing, cable and wire products industries, and solder and foundry work, which become the major source of lead contamination (1). Lead can be easily absorbed by the human body via the respiratory system and gastrointestinal tracts. It also has non-biodegradability and a very long half-lifetime (2). After absorption, lead is distributed in the blood, bone, and soft tissues. Lead interferes with cellular metabolism by interacting with the cellular macromolecules in tissues throughout the body, resulting in multisystem effects including hypertension, renal impairment, immunotoxicity, and toxicity to the reproductive organs (3). According to the World Health Organization (WHO) 2021 update, nearly half of the deaths attributable to chemical exposures in 2019 were due to lead exposure and resulted in cardiovascular diseases. Lead exposure is also estimated to be responsible for 30% of the global burden of idiopathic intellectual disability, 4.6% of the global burden of cardiovascular disease, and 3% of the global burden of chronic kidney diseases (4).

Heavy metal poisoning is usually treated with chelation by creating an insoluble, less toxic metal complex that can be easily excreted. According to the “Guideline for clinical management of exposure to lead” published by the WHO, Dimercaprol (British Anti-Lewisite, BAL), 2,3 -dimercaptosuccinic acid (DMSA), penicillamine, and sodium calcium edetate (CaNa_2_EDTA) were recommended as chelating agents for lead exposure treatment (5). Among them, the first three are sulfhydryl-containing compounds and the last is a hydroxyl-containing compound. CaNa_2_EDTA is most commonly used for the treatment of childhood lead poisoning. However, these chelators were reported to have several different safety and efficacy concerns. CaNa_2_EDTA therapy could cause essential metals such as zinc, iron, and manganese to be excreted and depleted because of its relative lack of specificity (6), some of which would even cause secondary damage to patients (7, 8). DMSA, approved by the US FDA, also has side effects such as gastrointestinal discomfort, skin reaction, mild neutropenia, and elevated liver enzymes (8). Therefore, screening for safe and effective lead removal drugs is a focus of current research. Some antagonistic drugs can also reduce the damage of lead to the body by enhancing the body's immunity or antioxidant capacity, such as beetroot juice and laver (9, 10). Ions such as calcium, iron, zinc, and magnesium have been also reported to have the ability to reduce the absorption of lead by competing with lead for intestinal absorption (11).

Edible fungi contain many pharmacological active ingredients, which have become a focus of much research as important resources for the development of functional food or drugs. Wild mushrooms have been reported to be highly susceptible to the enrichment of heavy metals in polluted areas (12), which indicates their strong affinity for heavy metals. However, the enrichment process depends on the environment polluted by heavy metals (13). Artificially cultivated mushrooms on non-polluted materials not only contain little heavy metal ions but also have the potential to be applied in lead poisoning therapy due to their metal affinity substances (14). In a previous survey of 35 kinds of commonly eaten edible fungi, *A. auricular* and *P. ostreatus* could reduce the lead content both *in vitro* and *in vivo*. In this study, we purified the active substance with lead absorption and evaluated its ability to reduce the lead level in rats' blood and tissues. These two kinds of mushrooms have been emphasized to be used for various biological activities such as antioxidative (15, 16), antimicrobial (17, 18), hepatoprotective (15, 19), and hypolipidemic (15, 20) action. This study suggests that these edible fungi could serve as new resources for developing antidotes for lead intoxication.

## Materials and methods

All experimental protocols were approved by the University Safety Office and Animal Experimentation Ethics Committee at China Agricultural University. All tests were conducted in accordance with the approved guidelines of the Animal Care and Use Committee of China Agricultural University.

### Materials

Fruiting bodies of *A. auricular* and *P. ostreatus* were purchased from a local market. Male Sprague-Dawley rats weighing 250 ± 20 g were supplied by Huafukang Biosource Bio Company, Beijing (SCKK 2014-0004). No significant differences in body weight exist among the animals used for the studies (*P* < 0.05).

### Determination of *in vitro* lead clearance ratio

For samples from the various purification steps, each of the fractions was dissolved in deionized water at a final concentration of 4 mg/mL. A mixture of equal volumes of a sample and 0.001% lead standard solution could be vibrated at 160 rpm for 3 h at room temperature. The control group was treated with deionized water instead of a contaminated sample. To precipitate the polysaccharides, a quadruple volume of ethanol was added to the reaction mixture which was vibrated and left to stand for 1 h. The supernatant was obtained by centrifuging at 9,000 rpm for 10 min. The supernatant was diluted appropriately for determining the concentration of lead by atomic absorption graphite tube spectrometry.

The clearance ratio was calculated as follows:


$$\begin{array}{l} \;Clearence\;ratio\left(\%\right)=\\ \frac{Concentration\;in\;control\;group\;-\;Concentration\;in\;experiment\;group}{\;concentration\;in\;control\;group}\times 100 \end{array}$$

Samples from rats, such as blood, liver, spleen, and kidney were subjected to microwave digestion before determination of the lead content. Blood samples (0.5 mL) or organ powder (0.5 g) along with HNO_3_ (5 mL) and H_2_O_2_ (2 mL) were placed in a digestion tank which was placed in a microwave digestion system. The program was started according to the manufacturer's instructions. Upon the completion of the protocol, the sample in the tank was diluted into 50 mL and used for the determination of the lead content using Z2000 Atomic Absorption Graphite Tube Spectrometry.

### Purification of AAAS and POAS

The crude active substances from *A. auricular* (AAAS) and *P. ostreatus* (POAS) were prepared using the following protocols: we dried the fruiting bodies to constant weight and crushed each specimen to powder; 100 g powder could be homogenized in deionized water (mass to volume ratio = 1:10) and incubated for 6 h at 95°C. After centrifugation at 9,000 rpm for 10 min, the sediment was repeatedly incubated with deionized water (*m*:*v* = 1:1) at 95°C for 3 h for better extraction. To obtain the water extract, supernatant from two centrifugation runs were combined and reduced the volume by using vacuum-rotary evaporation at 65°C. This water extraction was then alcohol precipitated by adding a quadruple volume of ethanol and standing overnight. The crude active substances cAAAS and cPOAS were enriched in the sediment after centrifugation of the mixture and then dried at 45°C and stored for future use.

By pre-experiment, the final protocols of AAAS and POAS purification were designed following the same three steps albeit with a slight difference in the second step. The first step is anion-exchange chromatography. Before loading on DEAE-cellulose (pH 7.0) that had been thoroughly pre-equilibrated with deionized water, cAAAS and cPOAS powder were dissolved in ddH_2_O and centrifuged at 9,000 rpm for 10 min, respectively. After sample loading, the column was eluted with 0 M, 50 mM, 150 mM, and 1 M NaCl sequentially, at a flow rate of 5 mL/min. The effluent fraction was collected in tubes and the concentrations of polysaccharide and protein were determined by using the phenol–sulfuric acid method (A490 nm) (21) and UV spectrophotometry (A280 nm), respectively. According to the elution plot, four fractions were collected separately. By checking the lead clearance rate *in vitro*, the fraction with the highest activities was collected for the next step. The second step involved ultrafiltration: the ultrafiltration membranes for AAAS and POAS were screened at 1 and 3 kDa, respectively, depending on their molecular weight. Both the solution and filtrate intercepted by the ultrafiltration membrane were freeze-dried in a vacuum, and the lead clearance was determined at different concentrations. Finally, the fraction possessing the higher lead clearance activity was then concentrated for gel filtration chromatography on a Superdex peptide 10/300 GL (GE Healthcare) or Superdex 75 10/300 GL (GE Healthcare) with an AKTA Purifier system. The buffer for loading and elution was deionized water (pH 7.0) and NH_4_HCO_3_ solution (pH 8.5), respectively. The purified AAAS and POAS were collected by lead clearance assay of the fractions eluted and freeze-dried into powder under a vacuum for future use.

### SDS-PAGE and sequence analysis of AAAS and POAS

Purified AAAS/POAS was dissolved in 20 μL of ddH_2_O and mixed with a 5×tricine-SDS-PAGE loading buffer. The mixture was cooled immediately after being boiled for 10 min. The sample was loaded on a Novex™ 10–20% tricine protein gel and electrophoresed at 120 V according to the manufacturer's instructions. The gel was then stained by Coomassie brilliant blue R-250, and de-stained by ethanol and acetic acid according to the manufacturer's instructions. The AAAS/POAS stripe was cut down and analyzed by LC-MS/MS for peptide sequencing.

### Preparation of reagents related to animal experiments and the drug delivery methods

Lead acetate solution (4 g/L) was prepared by dissolving 1 g Pb(Ac)_2_•3H_2_O into 250 mL 0.4% HNO_3_ solution. CaNa_2_EDTA (37.5 g/L) was prepared as a positive drug as it has a remarkable lead removal capacity. AAAS and POAS powder were dissolved into deionized water at a concentration of 12 mg/mL. The above reagent was stored at 4°C and placed at room temperature 2 h in advance. *A. auricular* and *P. ostreatus* fruiting body powders were mixed with basal feed powder and manually remade into fruiting body feed (AAFF and POFF) at a final concentration of 5%.

Rats were treated with lead through intraperitoneal (ip) injection of lead acetate solution. CaNa_2_EDTA, AAAS, and POAS were intragastrically (ig) administrated. AAFF and POFF were taken orally as a normal diet.

### Establishment and evaluation of the lead poisoning rat models

The SD rat models of acute lead poisoning were established by intraperitoneal injection of lead acetate solution at a dosage of 20 mg/kg body weight daily for 7 days in succession. The control (CTL) group was intraperitoneally injected with dilute nitric acid (0.4%) at the same volume. After 2 days of recovery, rats received deionized water through intragastric administration for 30 days and then anesthetized to death.

For the establishment of chronic lead-induced poisoning rat models, the rats were intraperitoneally injected with lead acetate solution for 7 days and recovered for 2 days (as aforementioned). Rats received a lower lead treatment (5 mg/kg body weight) in the following 30 days instead before death.

Body weights were recorded before and after lead treatment and on day 30. Blood samples of the rats were collected from the eye socket vein after rats were anesthetized every 6 days. At least 0.5 mL of blood from each rat was collected into a microextraction tube that contained heparin sodium and shaken repeatedly to avoid blood coagulation. All blood samples were preserved at −20°C.

### Removal of lead in SD rats that experienced constant lead poisoning

Thirty-five SD rats were divided into seven groups with five rats per group. The seven groups received different treatments (**Figure 4A**). Rats were anesthetized to death after the last blood collection. The liver and kidneys were collected, rinsed with saline, freeze-dried, and pulverized. Both blood samples and organ powders were nitrolyzed, and the concentration of lead was determined using atomic fluorescence spectroscopy.

### Statistical analysis

Data are shown as mean ± standard deviation (SD). Normality tests were run using the Shapiro–Wilks test in SPSS 22.0 software (IBM, Chicago, IL, USA). Different groups were compared by using a one-way analysis of variance (ANOVA) followed by the Tukey test using SPSS 22.0 software. *P*-values <0.05 were considered significant.

## Results

### Purification of AAAS and POAS

**Figure 1:**
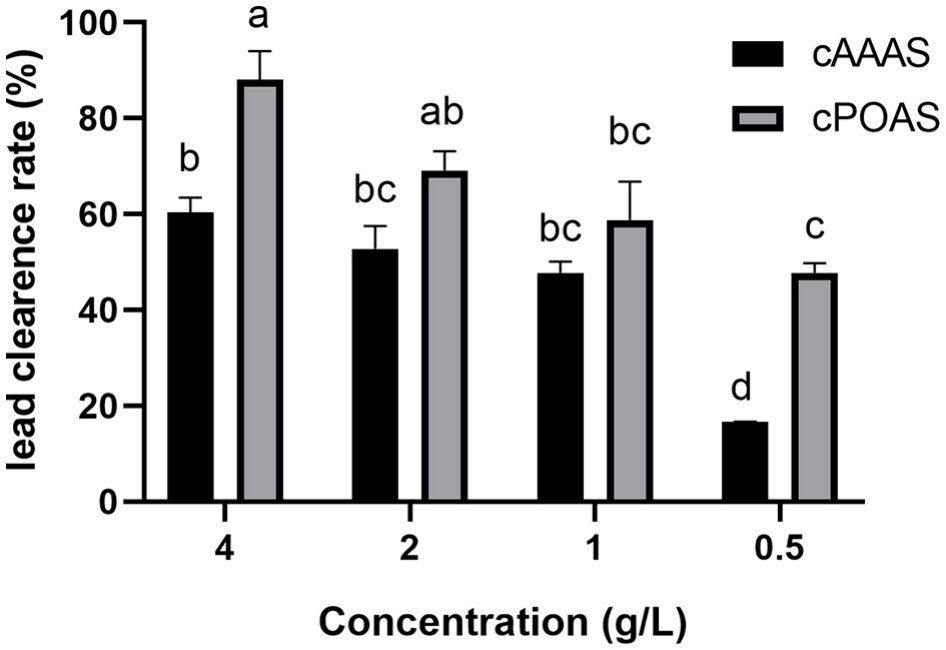
Lead clearance rate of different concentrations of cAAAS and cPOAS. Different letters indicate a significant difference (*P* < 0.05).

Through hot-water extraction, alcohol precipitation, and drying, crude active substances from the fruiting bodies of *A. auricular and P. ostreatus* (cAAAS and cPOAS) were obtained from the fruiting body with yields of 8.64 and 3.12%, respectively. cAAAS was presented as a white powder, while cPOAS powder was brown. The *in vitro* lead clearance activities of cAAAS and cPOAS were also determined at different concentrations (Figure 1). When combined with lead, the results showed that both cAAAS and cPOAS had a dosage-dependent removal effect of lead. The lead clearance rates of cAAAS at different concentrations as 0.5, 1, 2, and 4 g/L were 16.70, 38.46, 52.82, and 60.3%, respectively, while cPOAS clearance rates were 47.75, 58.82, 69.10, and 88.02%, respectively. The lead clearance rate of cAAAS was lower than that of cPOAS at the same concentration.

**Figure 2:**
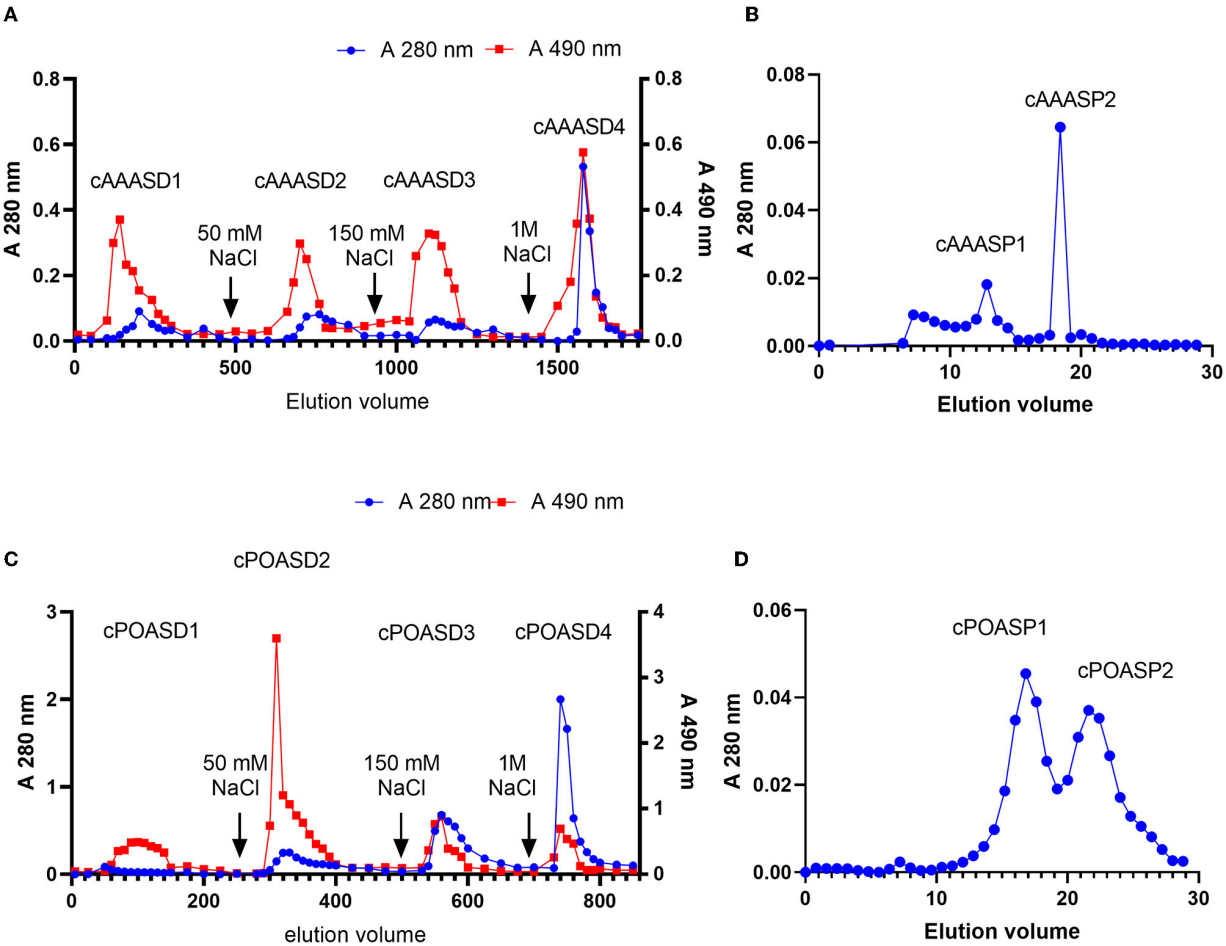
Elution curves of AAAS and POAS. **(A)** cAAAS on DEAE-Sepharose column (40 × 400 mm). **(B)** cAAASU2 on Superdex peptide 10/300 GL. **(C)** cPOAS on DEAE-Sepharose column (40 × 400 mm). **(D)** cPOASU2 on Superdex 75 10/300 GL.

A DEAE-Sepharose column was applied for the next purification step of cAAAS: four fractions (cAAASD1-cAAASD4) were collected by following the polysaccharide eluting curve, in which cAAASD2 possess the highest lead clearance activity (Figure 2A). To identify the molecular range of the active substance, an ultrafiltration membrane (1 kDa) was used to separate the cAAASD2 and two fractions named cAAASU1 (>1 kDa) and cAAASU2 (<1 kDa) were obtained. The two fractions had distinctively different lead removal activities (0.03 and 99.79%), indicating that the active substance has a lower molecular mass. Fraction cAAASU2 which possessed higher activity was then subjected to gel filtration on a Superdex peptide column and separated into two peaks (Figure 2B). The major lead clearance activity was enriched in the first peak (cAAASP1, i.e., purified AAAS), which possessed a molecular mass of about 4.9 kDa calculated from the standard protein filtration curve. In general, AAAS was purified 400-fold compared to the crude sample, and the recovery rate was 0.16% (**Table 2**).

On the other hand, there are also four fractions (cPOASD1-cPOASD4) obtained from the DEAE-Sepharose column when cPOAS was eluted (Figure 2C). The second fraction cPOASD2 showed the highest lead clearance activity. A 3-kDa ultrafiltration membrane was applied to separate the cPOASD2 into cPOASU1 (>3 kDa) and cPOASU2 (<3 kDa). Both the two fractions had lead clearance activity, but cPOASU2 was much higher than cPOASU1. The activities of several fractions under different concentrations are summarized in Table 1, from which we can see that when the reaction concentration was reduced below 0.04 g/L, most of the lead clearance activity of cPOASU2 was lost, whereas cPOASD2 could still maintain its high activity. This finding indicates that cPOASU1 may play an important role in maintaining the activity of cPOASU2. This speculation was further proved by the fact that the activity was restored after re-mixing the cPOASU1 and cPOASU2 under 0.04 g/L and 0.004 g/L. After gel filtration, cPOASU2 was also eluted in two fractions (Figure 2D) and the major activities enriched in the first peak (cPOASP1, i.e., purified POAS). Through the calculation of the lead clearance activity of cPOASU2, POAS was purified 10-fold compared to the crude sample, whereas cPOASD2 was 125-fold purified (Table 2). The most likely cause of this is the loss of component cPOASU1.

**Table 1:** Lead clearance activities of fractions in the purification step under different concentrations.

**Concentration (g/L)**	**4**	**1**	**0.4**	**0.04**	**0.004**
cAAAS	69.55% ± 5.81%^c^	38.33% ± 4.25%^d^	15.33% ± 3.28%^e^	1.06% ± 1.03%^e^	ND
cAAASD2	93.62% ± 2.76%^b^	61.79 ± 3.96^c^	19.22% ± 2.80%^e^	4.78% ± 1.77%^e^	ND
cAAASU2	99.12% ± 1.31%^a^	97.65% ± 1.13%^a^	86.53% ± 1.99%^c^	51.93% ± 4.31%^c^	0.42% ± 0.78%^d^
cPOAS	92.01% ± 4.21%^b^	60.21% ± 1.88%^c^	45.23% ± 4.17%^d^	1.26% ± 1.11%^e^	0.23% ± 0.11%^e^
cPOASD2	98.54% ± 1.26%^a^	91.48% ± 2.74%^b^	88.65% ± 2.32%^c^	77.32% ± 1.47%^b^	56.74% ± 4.22%^a^
cPOASU1	39.92% ± 3.01%^d^	8.39% ± 1.98%^e^	4.56% ± 1.58%^f^	ND	ND
cPOASU2	98.86% ± 0.19%^a^	96.22% ± 0.95%^a^	93.26% ± 2.31%^b^	18.09% ± 4.04%^d^	3.26% ± 2.25%^c^
cPOASU2+ cPOASU1	91.48% ± 2.74%^b^	97.90% ± 1.66%^a^	98.85% ± 0.38%^a^	93.49% ± 2.12%^a^	30.16% ± 1.29%^b^

All values are represented as mean ± SD (n = 3). Different letters in the same raw indicate a significant difference (*P* < 0.05).

**Table 2:** Efficiency of the active substances from *A. auricular and P. ostreatus*.

**Fraction**	**Concentration required to remove half of the lead (g/L)**	**Recovery rate (%)**	**Purification *n* (*n*-fold)**
cAAAS	2	100	1
cAAASD2	0.8	38.42	2.5
cAAASU2	0.04	6.88	50
AAAS	0.005	0.16	400
cPOAS	0.5	100	1
cPOASD2	0.004	22.33	125
cPOASU2	0.08	5.21	6.25
POAS	0.05	0.12	10

### Amino acid sequencing of AAAS and POAS

The ESI-MS/MS of AAAS revealed three peptides with higher reliability as follows: RAAELEAEREGAR, YEELQRVSR, and WLTTDEARFLR. The three peptides contained many polar amino acids accounting for 53, 77, and 55%, respectively, and all reached more than half. In addition, all three peptides contained closely related acid amino acids (aspartic acid or glutamic acid). The ESI-MS/MS of POAS demonstrated four peptides with high reliability and high homology with actin from *Wallemia ichthyophaga*, a salt-tolerant fungus. These peptides were: TTGIVLDSGDGVSHTVPIYEGYALPHAIIR, VAPEEHPVLLTEAPLNPK, SYELPDGQVITIGNER, and AVFPSIVGRPR. The proportions of polar amino acids were 40, 61, 56, and 55%, respectively.

### The establishment of acute and chronic lead-induced poisoning rat models

**Figure 3:**
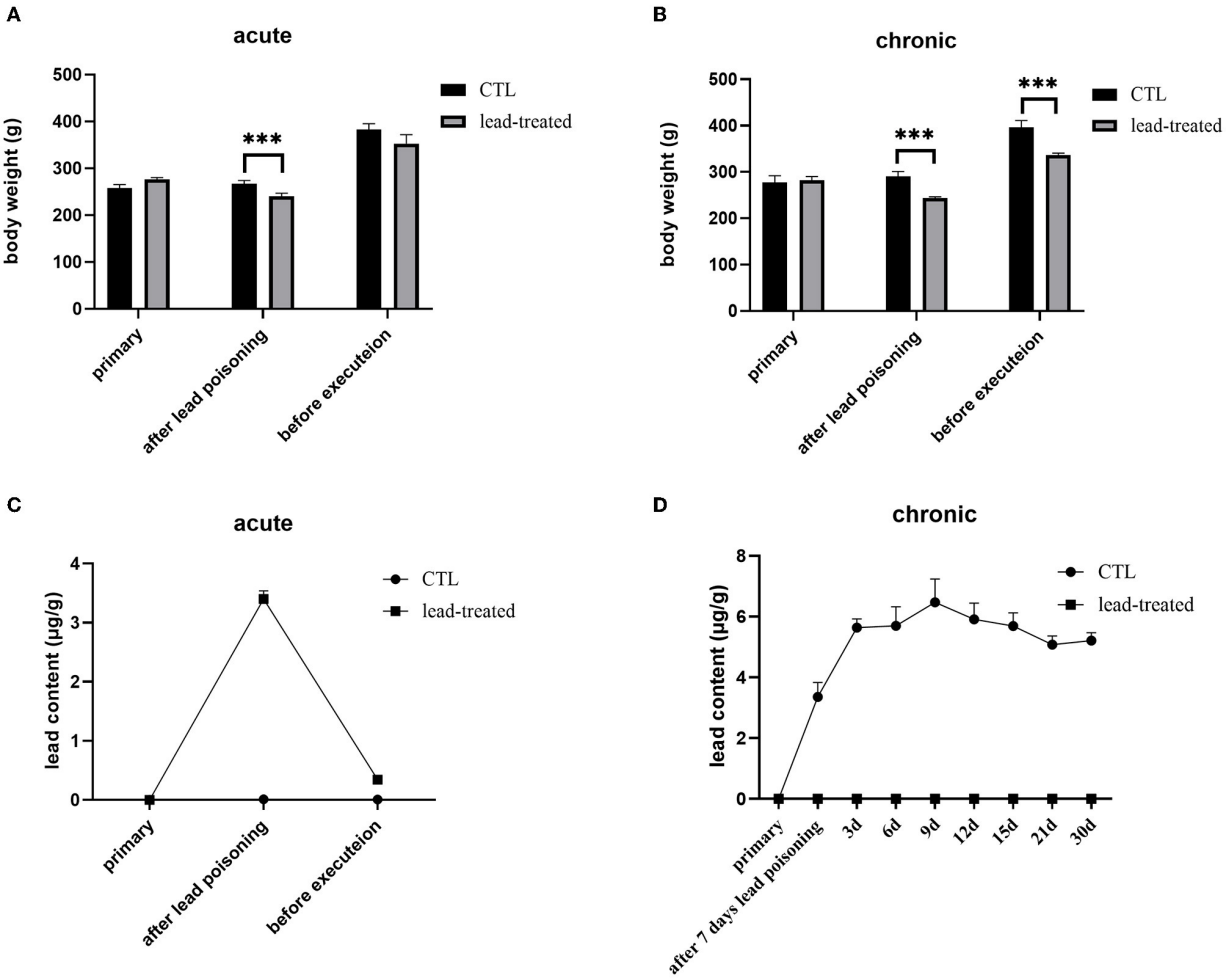
Acute and chronic lead-induced poisoning rat models. **(A, B)** were the bodyweight of rats subject to acute or chronic poisoning, respectively; **(C, D)** were the lead content in the blood of the rats subject to acute or chronic poisoning. All values are represented as mean ± SD (*n* = 6). ***P < 0.001 vs. CTL group as revealed by ANOVA.

In the process of lead poisoning, rats treated with lead have darker hair, slower reactions, and a reduced food intake compared with the rats in the control group. After lead treatment for 7 days, the rats' body weight decreased significantly (*P* < 0.001) compared with the CTL group both in the acute and chronic models (Figures 3A, B). In the acute poisoning model group, the body weight of rats receiving no more lead treatment in the subsequent 30 days recovered and was not significantly different (*P* < 0.05) from rats in the CTL group. Whereas, the body weight of rats in the chronic group could not recover to the levels in the CTL group. The lead content in blood was increased significantly after lead treatment for 7 days (*P* < 0.001). The lead content in blood decreased to normal levels with no more lead treatment in the acute poisoning model group whereas it increased continuously and cannot decrease to a normal level under low-dosage lead treatment in the chronic model group (Figures 3C, D). This result indicates that lead can affect the health of rats, but once free from lead exposure, rats are recovered from acute lead poisoning. Persistent lead exposure can continually affect the health of rats. The chronic lead poisoning group was more suitable for the current study.

### Detoxifying effect on chronic lead poisoning in rats

**Figure 4:**
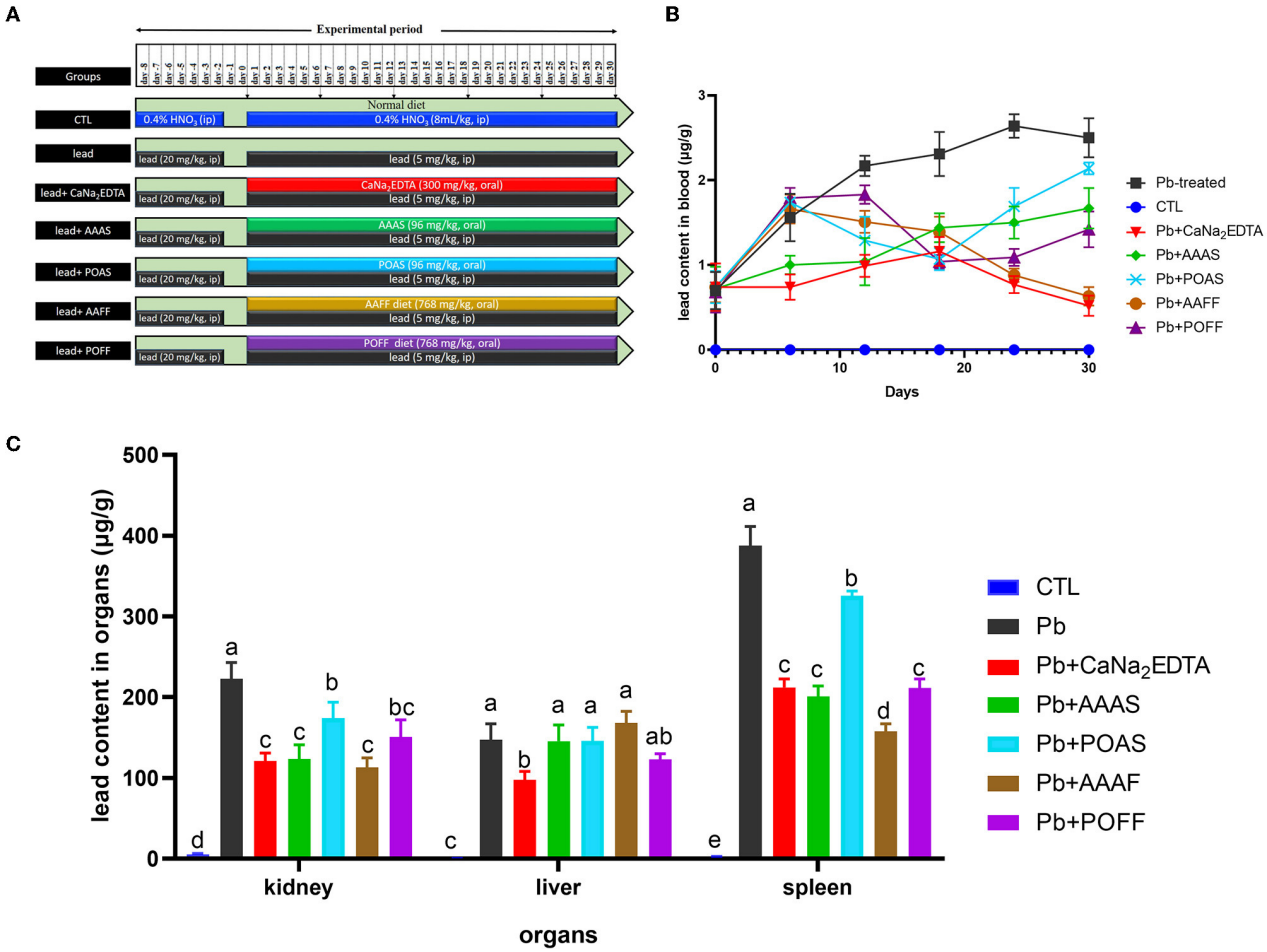
Elimination of lead in the blood and organs of rats. **(A)** Treatment of rats from different groups. **(B)** Lead contents in the blood sample of rats. **(C)** Lead contents in different organs of rats. All values are represented as mean ± SD (*n* = 5). Different letters for the same parameter are significantly different at *P* < 0.05 as revealed by ANOVA.

In the process of high dosage of lead, rats were unresponsive, showing reduced activity, loss of appetite, and reduced diet except for the control group: this body condition recovered after subsequent therapy compared to rats in the model group. The level of lead in blood is presented in Figure 4B. We also summarize the reduction rates of all treated groups compared to the lead-induced poisoning group (Table 3). Rats treated with (CaNa_2_EDTA) and AAAS had a significantly lower level of blood lead from day 6 to the end of the experiment period (*P* < 0.01 and *P* < 0.05). On day 30, the blood lead level decreased by 79.2 and 33.2%. The reduction of the blood lead level of POAS was displayed on day 12 and reached 14.4% at end of the treatment period (somewhat poorer than AAAS and CaNa_2_EDTA). Fruiting body feeds AAFF and POAF exhibited higher blood lead removal activities at the later period of treatment (from day 24) which were both better than the purified AAAS and POAS, indicating that fruiting body powder may play other roles in lead poisoning therapy.

**Table 3:** Average reduction rate (%) of the blood lead level compared with the lead-treated rats.

	**0 d**	**6 d**	**12 d**	**18 d**	**24 d**	**30 d**
Lead + CaNa_2_EDTA	ND	52.6	54.4	49.8	70.1	79.2
Lead + AAAS	ND	35.9	52.1	37.7	43.2	33.2
Lead + POAS	ND	ND	40.6	53.7	36.0	14.4
Lead + AAFF	ND	ND	30.4	39.8	66.7	74.8
Lead + POFF	ND	ND	15.7	55.0	58.7	43.2

The depth of the color in each row indicates the significant difference in the blood lead content compared with the lead-treated group: 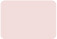 *P* < 0.05; 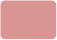 *P* < 0.01; 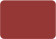 *P* < 0.001.

The lead contents in the kidney, liver, and spleen were also detected after the trial (Figure 4C). Compared with the CTL group, the lead contents in all three organs of rats in the lead-treated group were significantly increased (*P* < 0.001). After intragastric administration with CaNa_2_EDTA, the lead contents in the kidney, liver, and spleen were reduced by 45.7, 33.7, and 45.3%, respectively, compared with the lead-only treated group. Purified AAAS, POAS, and the fruiting body of these two mushrooms all had the capacity to reduce the lead content in the kidney and spleen to some extent but failed to do so in the liver. AAAS and AAFF exhibited a better lead clearance rate from the kidney and spleen than CaNa_2_EDTA.

## Discussion

*A. auricular* and *P. ostreatus* are two of the most consumed edible fungi, especially in China. Our laboratory has found that the crude polysaccharide of *A. auricular* and *P. ostreatus* can reduce the blood lead level of rats. This study further purified the substances with lead removal activity and then evaluated their lead removal activities both *in vitro* and *in vivo*.

The crude extracts of active substances cAAAS and cPOAS were obtained from AAAS and POAS fruiting bodies by water-extraction and alcohol precipitation methods as described (22). However, cPOAS had better lead removal activity than cAAAS *in vitro*. At a concentration of 4 g/L, cPOAS removed 88% of the lead, while cAAAS removed <70%, indicating that these two substances have different affinities to lead. The further purification protocols of AAAS and POAS were similar. The concentration required to remove half of the lead indicates the ability of lead clearence. As the purification steps increases, the concentration of the main faction requried to remove half of the lead decreases, evicing a growing lead-clearance ability. This means that the main substances that adsorbed lead were purified efficiently. Compared with cAAAS, the lead-removal activity of purified AAAS was nearly 400 times higher. Whereas, that of purified POAS was only 10 times than cPOAS. The loss of activity could have occurred after ultrafiltration when cPOASU1 was abandoned due to its weaker lead clearance activity. It is speculated that cPOASU1 may be an auxiliary substance of cPOASU2. When the two are combined, the full biological activity could be mobilized; however, the details of these two fractions need further investigation. Through the whole purification process, AAAS and POAS had absorbance under 280 nm and 490 nm through phenol–sulfuric acid assay, indicating that these components might consist of a polysaccharide and a peptide.

According to the molecular mass calculated by gel filtration chromatography of Superdex peptide, AAAS and POAS were 4.9 and 3.6 kDa, respectively. Both exceeded the interception molecular weight of the ultrafiltration membrane (1 and 3 kDa) but still penetrated. Most of the existing ultrafiltration membranes have a broad pore size (23), which will result in a broadband distribution of the membrane's intercepted molecular weight.

Amadi summarizes the mechanism of both heavy metal toxicity and antidote detoxification routes (24), pointing out that lead mainly caused systemic damage via the depletion of glutathione or bonding to sulfhydryl groups and accessible carboxyl groups of proteins. In addition, lead could cause oxidative pressure indirectly. The natural antidotes of heavy metals were usually worked by inhibiting oxidation or acting as chelators. Most of the classic metal chelators such as D-penicillamine, DMSA, BAL, and DMPS contain thiols that competitively binding lead (8). In this study, the peptide chains identified in the purified AAAS and POAS all contain a large proportion of amino acids whose side chains contain hydroxyl, carboxyl, carbonyl, sulfhydryl, and amidogen. These functional groups have been reported to be heavy metal binding sites (25). There were also studies that revealed that microbial exopolysaccharides are highly capable of binding with metal ions due to the presence of numerous binding sites of negatively charged functional groups associated with proteins and polysaccharides (26, 27).

The *in vivo* lead clearance effects of AAAS and POAS were testified by employing lead intoxication rat models. Two lead-induced poisoning rat models were established in this study: acute poisoning and chronic poisoning. However, after leaving the lead exposure environment, the acute lead poisoning rats gradually recovered to health without treatment. Flora and Pachauri suggest that, in the basic principles of metal toxicity management, the first and most important step is the prevention of further metal absorption into the system (8). They described how a normal excretory system may expel metals to provide a gradual recovery from mild toxicity which is in accordance with our study.

The chronic lead-induced poisoning rat models may simulate intoxication cases resulting from occupational exposure or exposure due to lifestyle and were used to evaluate the function of AAAS/POAS and the fruiting bodies of the two mushrooms. In this study, purified AAAS and POAS exhibited clearance effects on blood, kidney, and spleen, which indicated their potential to be developed as antidotes for lead.

For the natural antidotes of lead, no more than 10 have been reported. Sharma et al. proved that extracts from garlic could protect male mice from lead-induced toxicity (28). Zhai et al. summarized this protective property against lead toxicity, finding that it could be attributed to not only its antioxidative ability but also its chelation ability. In addition to its sulfur-containing amino acids, S-allyl cysteine and S-allyl mercaptocysteine could prevent the intestinal absorption of lead. Treatment with green tea extract has been reported to decrease blood lead levels and lead-induced neurotoxicity (29), and Zhai et al. stated its detoxification effect due to its active constituent, catechins (30). Sesame oil has been reported to protect against lead-plus-lipopolysaccharide (LPS)-induced hepatic damage (31). Thiol-containing peptides, investigated by Ding et al., were hydrolysates from soy protein, showing high sequestering abilities for heavy metals including lead, and could act as natural detoxicants. Their binding affinity is not only affected by the concentration of the sulfhydryl group but also by the molecular weight distribution (32). The oral administration of tomatoes has been shown to reduce the accumulation of heavy metals involving cadmium, lead, and mercury in rat liver (33). *Moringa oleifera* have been confirmed to have ameliorating effects of lead-induced toxicities in the liver, kidneys, and blood (34). Flora et al. have reported that curcumin (15 mg/kg) could protect rats against lead-induced toxicity in the blood, liver, kidney, and brain with co-administration of lead acetate (25 mg/kg) by chelation (35). Compared to their result, in our study, 5 mg/kg AAAS or POAS could protect rats from lead-induced (20 mg/kg) induced toxicity.

With respect to edible fungus, few researchers have focused on their heavy metal detoxification activities. The methanolic extract of *P. ostreatus* can protect female rats against cadmium-induced nephrotoxicity (36). Our previous research demonstrated that *Grifola frondosa* and its polysaccharide-peptide could accelerate mercury excretion in the blood, liver, and kidney (22). The aqueous extract of *Ganderma lucidum*, a famous medicinal mushroom, could significantly reduce the amount of lead in the liver (*P* < 0.01) and kidneys (*P* < 0.05), but not in the spleen (37). Whereas, our result indicated that the lead removal effect was manifest in the kidney and spleen but not in the liver. However, *G. lucidum* is an expensive Chinese medicinal material and it has a bitter taste. Our results demonstrated that feeding with fruiting bodies of AAAS and POAS also has a curative effect in cases of lead poisoning. Despite the lead chelator being contained in the fruiting bodies, we speculate that these fruiting bodies may play antioxidant roles as proved elsewhere (38–41). Our research provides dietary strategies for the treatment of lead toxicity. Since both *A. auricular* and *P. ostreatus* are delicious mushrooms, they could be taken as a daily dietary supplement for a lead-exposed population to avoid lead accumulation.

## Data availability statement

The original contributions presented in the study are included in the article/supplementary material, further inquiries can be directed to the corresponding authors.

## Ethics statement

The animal study was reviewed and approved by University Safety Office and Animal Experimentation Ethics Committee at China Agricultural University.

## Author contributions

HW contributed to the conception and design of the study. WZ contributed by collecting and analyzing the data. XZ contributed by performing the experiment. XC and XJ contributed by preparing materials and reagents. GZ contributed to the writing and revising of the manuscript. All authors contributed to the manuscript revision, read, and approved the submitted version.
